# First clinical experience using a novel automated mapping algorithm for mapping of ventricular arrhythmias

**DOI:** 10.1007/s00380-023-02245-w

**Published:** 2023-02-14

**Authors:** Guram Imnadze, Philipp Sommer, Thomas Fink, Mustapha El Hamriti, Moneeb Khalaph, Martin Braun, Vanessa Sciacca, Khuraman Isgandarova, Denise Guckel, Christian Sohns

**Affiliations:** Clinic for Electrophysiology, Herz- und Diabeteszentrum NRW, Ruhr-Universität Bochum, Bad Oeynhausen, Germany

**Keywords:** Ventricular tachycardia, Catheter ablation, Automated mapping, Arrhythmia substrates, Personalized medicine

## Abstract

A new automated vector-based mapping algorithm (AMA) for 3-dimensional (3D) mapping has been introduced. The aim of this study was to present our experience using AMA to recognize additional catheter ablation targets in patients with ventricular arrhythmias (VA). A total of 16 patients (ICM; ischemic cardiomyopathy, *n* = 6; NICM; non-ischemic cardiomyopathy *n* = 10) suffering from VA underwent catheter ablation. Following bipolar voltage mapping, AMA was utilized to reveal zones of decelerated conduction velocity vectors (CVV) and this information was superimposed onto the 3D reconstructions and compared with the presence of scar. Mapping time was 28.1 ± 10 min for the endocardial reconstruction of the left ventricle (LV) and 17 ± 5.4 min for the epicardium (*n* = 6 patients). The mean area of LV low voltage was 13.9 ± 15% (endocardial) and 11.9 ± 5.7% (epicardial). Decelerating CVV zones were revealed in all patients (mean conduction velocity threshold of 39.3 ± 13%). Sustained VA have been terminated through ablation and substrate modification was performed in all patients. Correlation between the presence of CVV deceleration zones and areas of abnormal low voltage from bipolar mapping was revealed in only 37.5% of patients, but there was good correlation between scar from unipolar voltage mapping and the presence of CCV deceleration zones (94%; *p* = 0.008). The novel AMA may improve the understanding of individual VA substrates due to the visualization of decelerated CVV zones and their correlation with abnormal low voltage predominantly from unipolar mapping.

## Introduction

A new automated mapping algorithm (AMA) for 3-dimensional electroanatomical mapping (CARTO PRIME, BiosenseWebster Inc., Diamond Bar, CA, USA) has recently been introduced (Coherent®; BiosenseWebster Inc., Diamond Bar, CA, USA). This novel feature aims to visualize the electric-wave propagation over the myocardium by means of color-coding and direction vectors [1, 2], to improve the understanding of the individual arrhythmia mechanisms and substrates. The algorithm includes the visualization of areas with conduction/propagation barriers, in particular, slow or non-conducting (SNO) zones, as well as representative vectors for conduction velocity. AMA can also be used in a combination with conventional activation mapping in sinus rhythm and during sustained arrhythmia. The novel AMA has already made a remarkable contribution to improve and facilitate ablation procedures for complex atrial tachyarrhythmias [3], but only very limited data are available about its utilization and impact on the treatment of ventricular arrhythmias (VA).

Ventricular tachycardia (VT) is a major health issue in patients with structural heart disease and myocardial scar as well as fibrosis play a crucial role in the genesis and maintenance of VAs. The coexistence of surviving myocardial fibers within scarred fibrotic tissue leads to the formation of slow conduction zones and to a dispersion of activation and refractoriness which contributes to a milieu for tachycardia circuits. AMA might have the potential to discriminate corresponding regions of interest within the scar tissue or at the border zone between healthy and scarred tissue to assist VT ablation and to define potential additional ablation targets.

The aim of this proof of concept manuscript was to present our initial experience using AMA to guide the mapping process in patients with VAs. AMA was utilized to visualize potential ablation targets beyond the arrhythmia substrate from ultra-high-density mapping in multiple VT etiologies. The novel mapping approach aimed to improve the understanding of the slowest conduction zones during sinus rhythm or right-ventricular pacing according to an adjusted conduction velocity vector analysis.

## Materials and methods

### Mapping and ablation

This prospective observational study included consecutive patients undergoing VA ablation guided by CARTO PRIME and the novel AMA at our center between February 2020 and June 2021. All patients signed a written informed consent. Prior to ablation, all patients received transthoracic echocardiography to rule out LV-thrombus formation. Transesophageal echocardiography (TEE) was performed in patients with elevated risk for left atrial (LA) or LA appendage (LAA) thrombus. The ICD/CRT devices were interrogated prior to ablation and VA therapies have been deactivation during the ablation procedure.

Catheter ablation was performed under general anesthesia. Two diagnostic catheters were introduced via the femoral veins and positioned in the coronary sinus (6 Fr, Webster®, Biosense Webster, Inc., Diamond Bar, CA, USA) and the right ventricle (RV) (5 Fr, Webster®, Biosense Webster, Inc., Diamond Bar, CA, USA). For an antegrade approach, venous access was obtained via the right femoral vein. A single transseptal puncture was performed under fluoroscopic guidance using a modified Brockenbrough technique and an 8.5-Fr transseptal sheath (ViziGo, large curve, Biosense Webster, Inc., Diamond Bar, CA, USA) and an additional retrograde access to the LV was obtained via the right femoral artery.

Endo- and epicardial mapping was performed using a 3D-mapping system (CARTO PRIME®, Biosense Webster, Inc., Diamond Bar, CA, USA) and a multipolar mapping catheter (PentaRay®, Biosense Webster, Inc., Diamond Bar, CA, USA). Ultra-high density electroanatomical reconstruction of the RV and LV was conducted aiming for > 1000 mapping points. A low-voltage area suggestive for endocardial myocardial scar was defined as endocardial bipolar voltage of 0.1–1.5 mV. A low-voltage area suggestive for epicardial myocardial scar was defined as epicardial bipolar voltage of 0.1–1.5 mV and endocardial unipolar voltage of 5–8 mV. Tissue Proximity Indication applied in all cases of LV and RV mapping.

The amount of LA low voltage (from uni- and bipolar mapping) was measured using the area measurement tool. For the epicardial approach, access was gained via a subxiphoid anterior puncture using a micropuncture needle followed by the introduction of a guidewire. Afterwards a steerable sheath (Agilis Epi, St. Jude Medical) was inserted into the epicardial space. Prior to ablation, the course of the left-sided phrenic nerve was constituted using pacemapping.

Following bipolar voltage mapping, the AMA algorithm was utilized to reveal zones of decelerated conduction velocity vectors (CVV) based on manual thresholding enabling the dynamic view function. The novel AMA has been previously described in detail [1–4]. The threshold caliper for conduction velocity vector (Coherent®—visualization setup) was adjusted until the first slow conduction velocity vector (the bold arrows) appeared inside the electroanatomical map. Afterwards, the information from AMA was superimposed onto the endocardial reconstructions and compared with the presence of abnormal uni- and bipolar voltage suggestive for ventricular scar tissue or fibrosis. In case that slow CVV zones were located in a close relationship to areas suggestive for scarred myocardial tissue, we classified this as a match.

Radiofrequency current (RFC) was delivered in the power-controlled mode with a maximum power of 40 W, a maximum temperature of 43 °C, and a flow rate of 30 mL/min using an open-irrigated tip-ablation catheter (ThermoCool SmartTouch SF®, Biosense Webster, Inc., Diamond Bar, CA, USA). LV and RV ultra-high-density mapping was conducted as described above followed by programmed ventricular stimulation when the patient was not in sustained VT at the beginning of the procedure. When VA was sustained and hemodynamically tolerated, activation map was conducted, and critical isthmus sites were identified using entrainment mapping. If no sustained VA was inducible, comprehensive substrate ablation was performed targeting all low-voltage areas, as well as abnormal electrograms such as local abnormal ventricular activity (LAVA) and late potentials (LP). After ablation, pericardial effusion was ruled out and patients were monitored at our intensive care unit (ICU) for more than 24 h. The ICD/CRT device was reactivated before hospital discharge.

### Follow up

Patients were routinely followed up in our outpatient clinic at 3, 6 and 12 months. In addition, continuous device (ICD/CRT) interrogations were performed via telemedicine. At each follow-up visit, symptom-specific interviews, physical examination, echocardiography and on-site ICD/CRT interrogations were performed. In case of recurrent VA following catheter ablation, all episodes were obtained and analyzed by the treating physicians and redo-ablation was scheduled if applicable.

### Statistical analysis

Statistical analysis was performed using SPSS for Windows (Version 19.0, SPSS Inc., Chicago, IL, USA). We performed descriptive statistics using Mann–Whitney U-test and chi square test. The values are given as mean and Standard deviation. A *P* value smaller than 0.05 were consider significant.

## Results

### Patient characteristics

A total number of 16 male patients with a median age of 66.1 ± 12.4 years suffering from VA underwent catheter ablation in our hospital. The cohort consisted of six patients with history of ischemic cardiomyopathy (ICM) and ten patients with non-ischemic cardiomyopathy (NICM). All patients were scheduled for VT ablation due to drug-refractory ventricular arrhythmias and/or electrical storm and all patients have had a history of repetitive ICD therapies. No previous VA ablation was performed. At the time of ablation, all patients were on ongoing optimal antiarrhythmic drug therapy. The mean LVEF at the time of ablation was 34.3 ± 13.9%. All patients have had an ICD (*n* = 7 transvenous ICD; *n* = 1 subcutaneous ICD; *n* = 8 CRT-D). The patients’ baseline characteristics and are summarized in Table 1.

**Table 1:** Patient baseline characteristics

	NICM *n* = 10	ICM *n* = 6	*P* value
Age *Y*	64	69.5	0.6
CRT%	60	33	0.32
EF%	36	32	0.49
Hypertension%	20	66	0.12
Diabetes%	20	33	0.6
CKD%	30	50	0.61
AF%	70	33	0.3
TIA%	40	16	0.59
COPD%	10	16	1

*CRT* cardiac resynchronization therapy; *EF* ejection fraction; *CKD* chronic kidney disease; *AF* atrial fibrillation; *TIA* transient ischemic attack; *COPD* chronic obstructive pulmonary disease

### Procedural data and follow-up

Mean procedure duration (skin-to-skin) was 160 ± 26.9 min, mean fluoroscopy time was 11.8 ± 6.2 min. and mean fluoroscopy dose was 2.078 ± 2.749 *γ*Gym^2^. During mapping, a mean of 2.058 ± 1.159 points were acquired. Mean mapping time was 28.1 ± 10 min for the endocardial reconstruction of the LV and 17 ± 5.4 min for the epicardium (*n* = 6 patients). The mean area of low-voltage was 13.9 ± 15% from endocardial mapping and 11.9 ± 5.7% from epicardial mapping. Decelerating CVV zones were revealed in all patients using a mean conduction velocity threshold of 39.3 ± 13%. The individual distribution of low voltage suggestive for scarred tissue and their relationship with areas of slow conduction from AMA is demonstrated in Table 2.

**Table 2:**
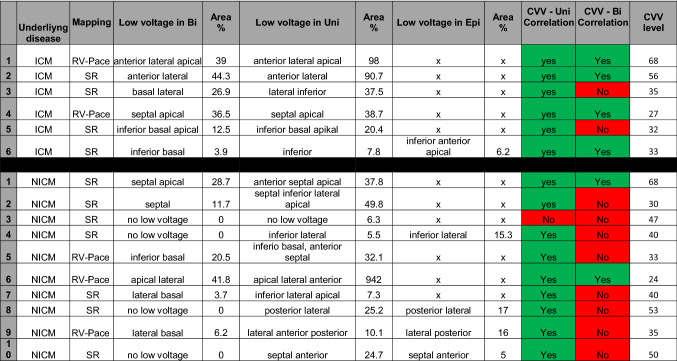
The procedural information of the scar location, scar area and CVV match in all patients

The correlation between CVV and low-voltage areas is shown in green. No correlation between CVV and low-voltage areas shown in red

*ICM* ischemic cardiomyopathy, *NICM* non-ischemic cardiomyopathy, Bi bipolar voltagemap, Uni unipolar voltagemap, *Epi* epicardial voltagemap (bipolar), Low voltage—Anatomical localization of abnormal (low) voltage. *CVV* conduction velocity vector, Mapping Heart Rhythm during mapping, *RV* right ventricle, *SR* sinus rhythm

Substrate modification targeting all late potentials and local abnormal ventricular activation (LAVAs) in scar border zones were performed in all cases. The findings from AMA were also integrated into the diagnostic process when there was its match with low-voltage area from uni- or bipolar high density mapping. Mean radiofrequency (RF) ablation time was 39.8 ± 16 min. We observed no procedure related complications requiring intervention. After follow-up (16 ± 5.1 months) three patients from NICM group (30%) and two patients from ICM (33%) group had VT recurrence.

### Correlation of CVV and low-voltage areas

**Fig. 1:**
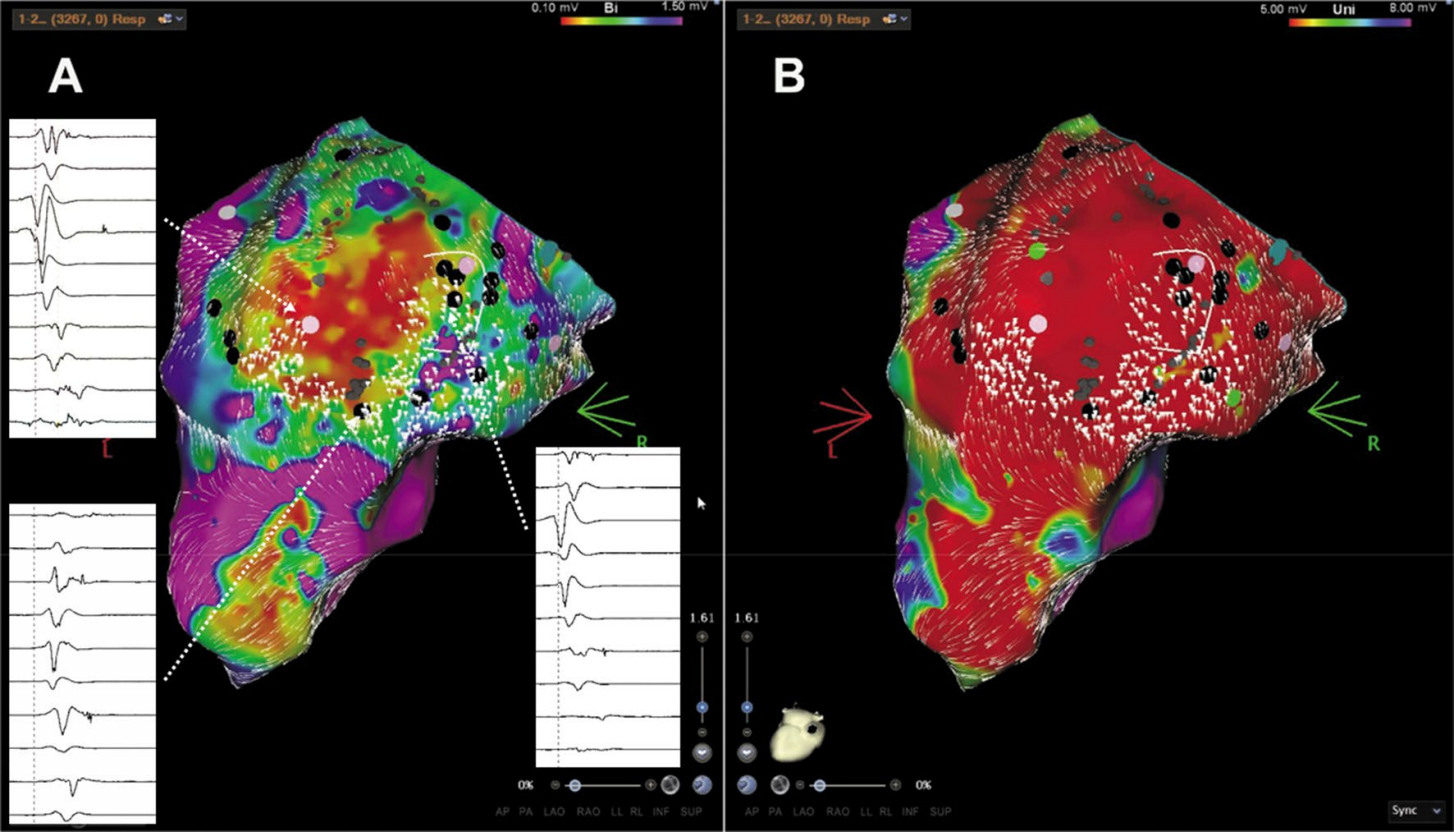
High density mapping with information from endocardial **A** bi- and **B** unipolar voltage discriminating a huge basal inferior and inferior-apical scar area in a patient with ischemic cardiomyopathy (ICM). The decelerated CCV areas are around the endocardial core of the scarred myocardium. Black dots depict potential VT channels at the scar border and pink dots depict LAVAs. **B** endocardial unipolar voltage map (5–8 mV) shows an extended low-voltage area compared with bipolar mapping and the area of decelerated conduction is located inside the scar tissue. Correspondence of LAVA and CVV areas. *CVV* conduction velocity vector; *LAVA* local abnormal ventricular activation

**Fig. 2:**
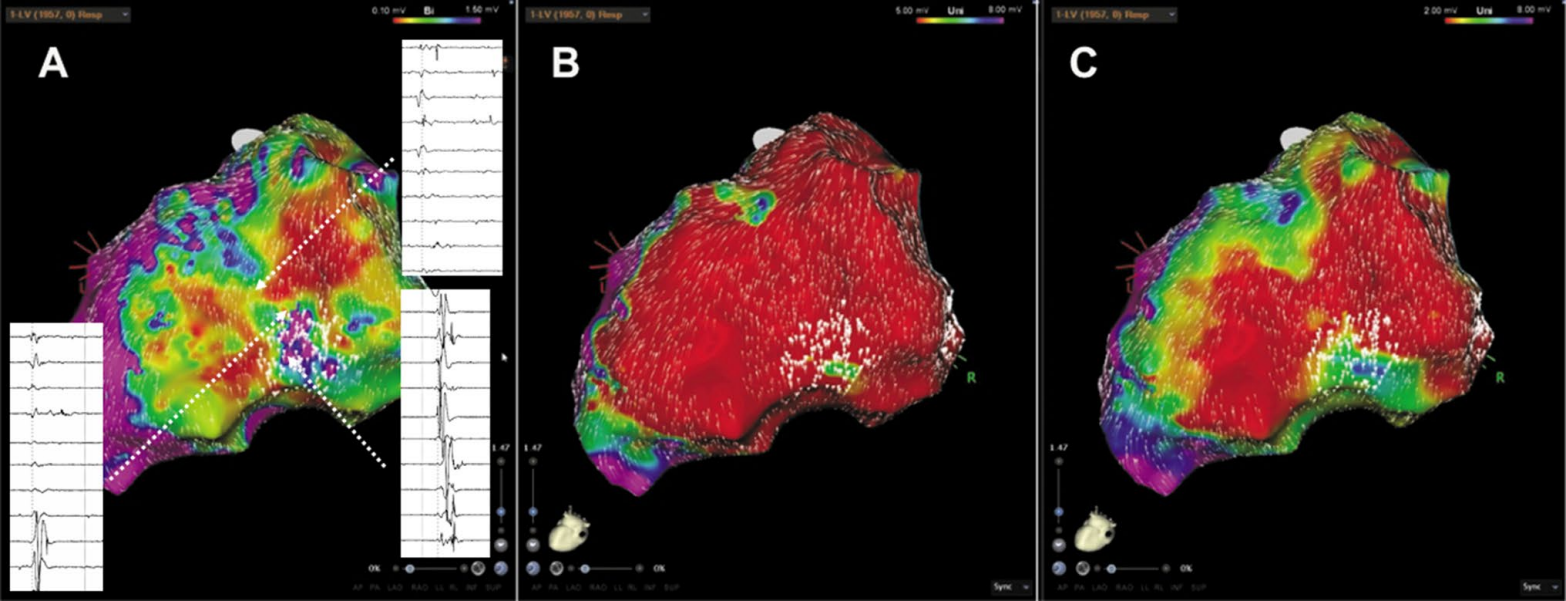
Typical example of a patient with ischemic cardiomyopathy. **A** Bi- and **B, C** unipolar endocardial LV reconstructions. **A** Endocardial bipolar voltage mapping (reference interval: 0.1–1.5 mV) shows a huge arrhythmia substrate affecting the inferior wall. Areas of decelerated CVV are located close to the scar border zone but still at a site with normal voltage. **B** Endocardial unipolar voltage mapping (reference interval: 5–8 mV) revealed an extended inferior scar, also suggestive for a relevant epicaridal scar. After the adjustment to unipolar voltage, the deceleration CVV zone is inside the scar. **C** Endocardial unipolar voltage reconstruction with a lower threshold (2–8 mV) shows a scar shape comparable to the bipolar distribution of abnormal voltage and the slow conduction areas are exactly around the dense scar borders. Moreover, it was also the place of the critical isthmus of the VT circuit in this patient. Correspondence of LAVA and CVV areas. *CVV* conduction velocity vector; *LAVA* local abnormal ventricular activation

**Fig. 3:**
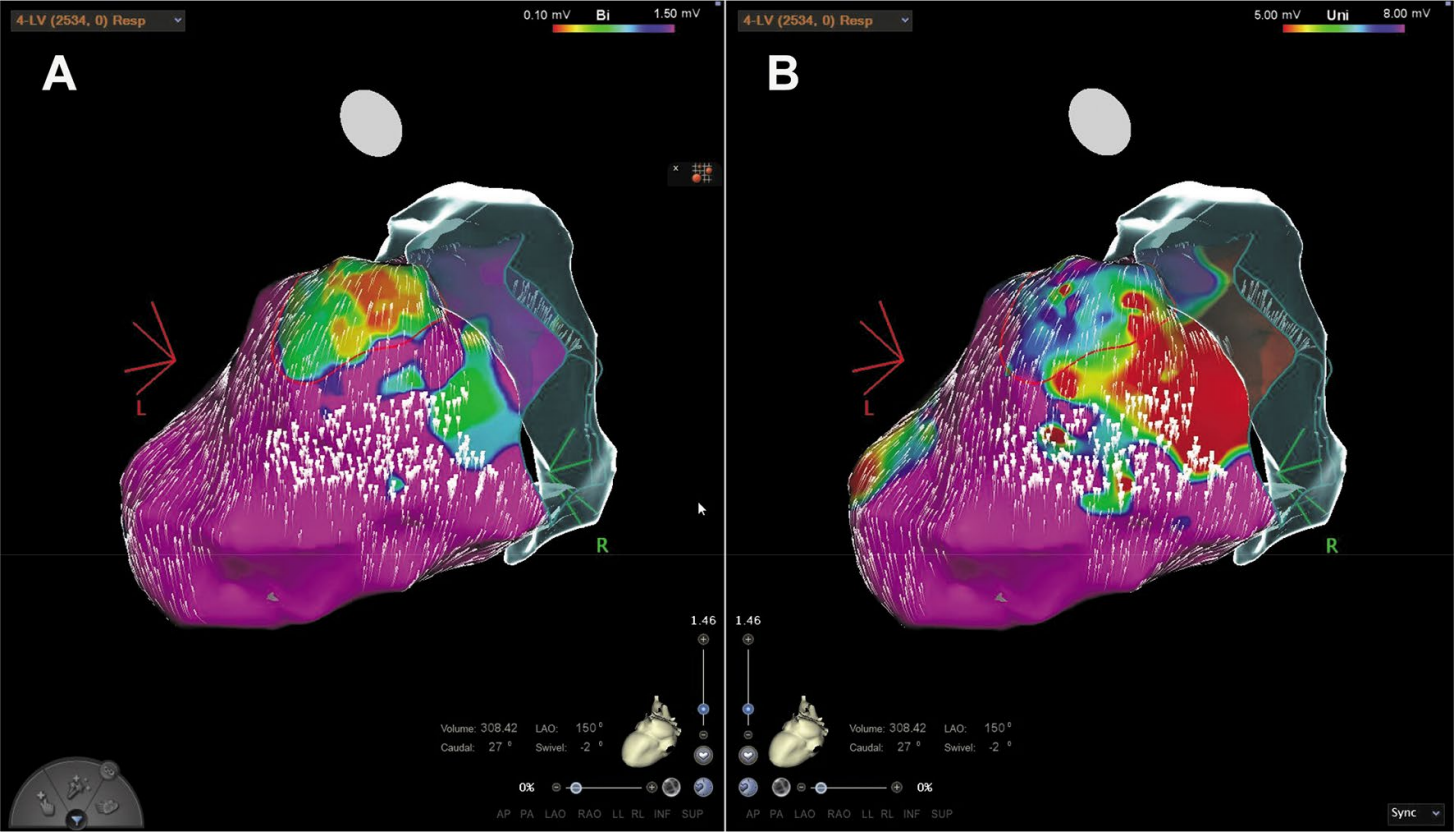
**A** Bi- and **B** unipolar endocardial reconstructions of the LV in a patient with dilated cardiomyopathy. **A** Endocardial bipolar voltage mapping (0.1–1.5 mV) highlights a small circumscribed scar area on the basal lateral wall. The areas of slowest conduction are located in an area suggestive for normal/ healthy myocardium.. **B** Using the information from unipolar mapping (reference interval: 5–8 mV) with an extended scar area, results in a perfect match between scar tissue and the findings from AMA

**Fig. 4:**
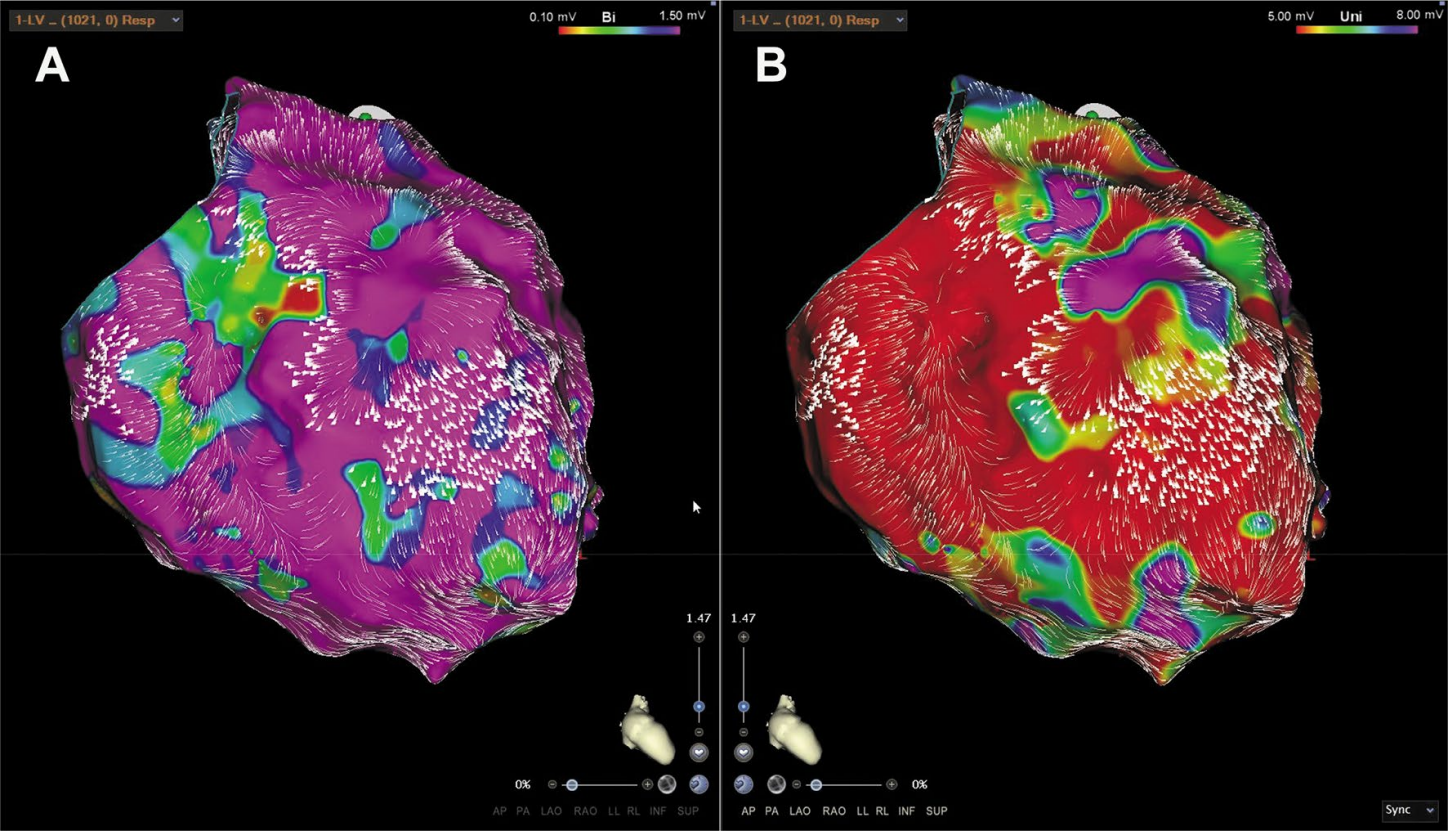
Representative example of the correlation between the findings from AMA and scar tissue from uni- and bipolar low-voltage mapping in a patient with dilated cardiomyopathy.. **A** Endocardial bipolar voltage mapping (reference interval: 0.1–1.5 mV) shows small and diffuse low-voltage areas at the infero-septal wall. The slowest conduction areas from AMA are located at site of normal voltage. **B** Endocardial unipolar voltage mapping (reference interval 5–8 mV) visualized a huge arrhythmia substrate at this anatomical site. The slow conduction areas are located along the border zone and inside the scarred myocardium

**Fig. 5:**
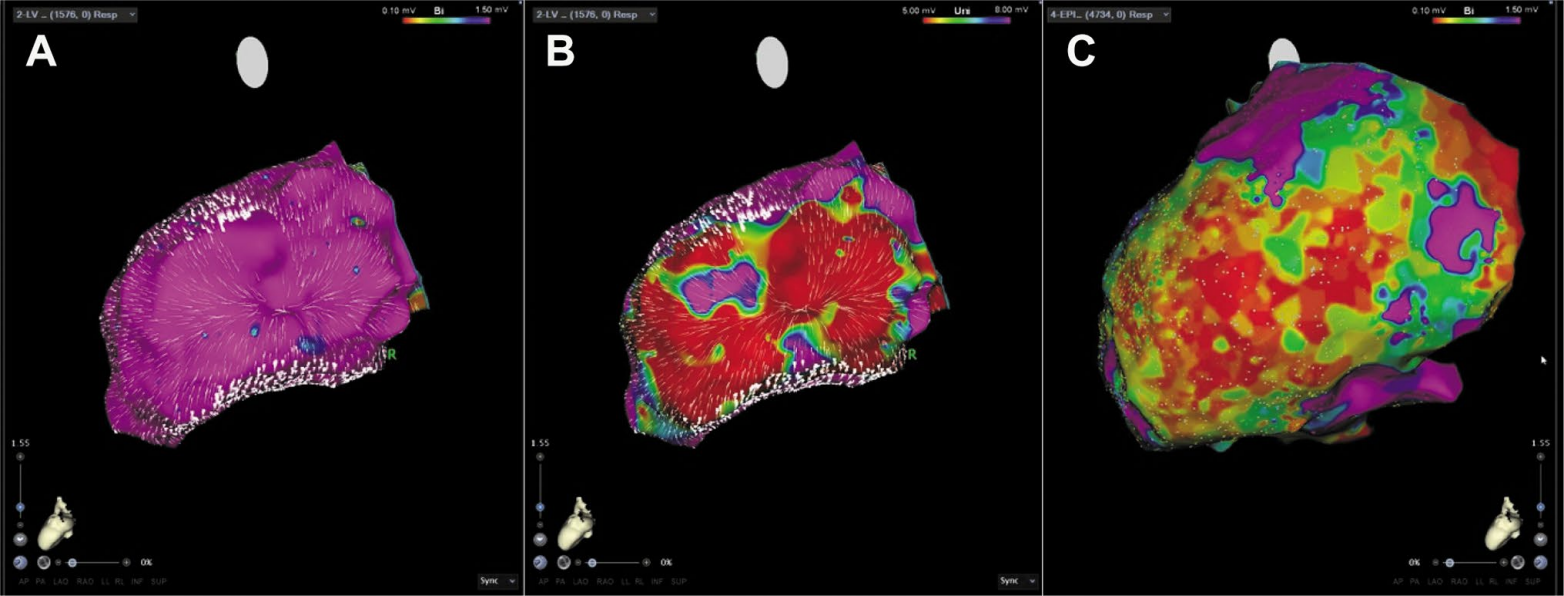
Endo- and epicardial LV reconstructions show a good correlation between scar tissue and deceleration zones from AMA in a patient with dilated cardiomyopathy. **A** Endocardial bipolar voltage mapping (reference interval: 0.1–1.5 mV) shows no evidence for an arrhythmia substrate but two deceleration areas. **B** Endocardial unipolar voltage mapping (reference interval: 5–8 mV) highlights a posterolateral arrhythmia substrate suggestive for epicardial scar tissue. Of note, the slow conduction areas are exactly at the border zones. **C** Epicardial bipolar voltage mapping (reference interval: 0.1–1.5 mV) reveals a huge scar correlating with the findings from AMA

The correlation between CVV deceleration zones from AMA and areas of abnormal low-voltage from endocardial uni- and bipolar mapping was analyzed as follows:

The individual distribution of low-voltage areas from endocardial unipolar mapping correlated significantly more often with zones of decelerated CVV (unipolar voltage mapping/ CVV analysis 94% vs. bipolar voltage mapping/ CVV analysis 37.5%; *p* = 0.0008), (Table 2.). In addition, we also found a better correlation between arrhythmia substrates from endocardial bipolar low-voltage mapping and decelerated CVV zones in patients with ICM as compared to those with DCM, As demonstrated in Fig. 1, the decelerated CVV zones were revealed in the center of a circumscribed low-voltage area from bipolar mapping potentially representing the focal “core area” of abnormal electrical activation and propagation in ICM related scarred tissue. Another observation was the presence of LAVAs and “channels” at anatomical locations with a good match between substrates from low-voltage and decelerated CVV zones (Fig. 1, Fig. 2, Fig. 3, Fig. 4, Fig. 5).

Despite these findings inside the scarred arrhythmia substrate, additional zones of decelerated CVV haven been observed also in close relationship to the border area between normal and scarred myocardium especially in endocardial bipolar maps of ICM patients (Fig. 2). In DCM patients, we frequently observed decelerated CVV zones at anatomical locations without any evidence for scar/ fibrosis from endocardial bipolar voltage mapping but from unipolar mapping (Fig. 3, Fig. 4, Fig. 5). In five patients, endo- and epicardial mapping and ablation were performed. In all of these patients, the distribution of abnormal low-voltage and decelerated CVV zones correlated well with the presence of low voltage from epicardial bipolar mapping (Fig. 5). In only one patient, no correlation was found between the CVV analysis and high-density mapping, as there was no evidence for the presence of low voltage at all.

## Discussion

This study has following major findings: first, AMA mapping has been easily integrated into a routine workflow and appears to be useful. Second, areas of decelerated CCV have been revealed in all patients. Third, distribution and location of decelerated CCV areas differ in patients with ICM and DCM. Fourth, correlation between the findings from AMA and the individual arrhythmia substrate was superior when utilizing the information from unipolar mapping.

Recently, Anter et al. demonstrated, that AMA resulted in improved identification of atrial arrhythmia substrates and origin in terms of focal and localized reentry. AMA also identified a higher number of localized reentrant circuits that were incorrectly identified as focal tachycardias by the standard activation mapping algorithms [1]. This is in line with findings from Vicera et al., who reported that AMA with CVV significantly improved the identification of critical arrhythmia isthmus sites in scar‐related atrial tachycardia [2]. One might speculate, that AMA might facilitate the recognition of slow conduction areas and critical sites representing potential targets of ablation, when used in conjunction with conventional mapping approaches. Only very limited data exist about the impact of AMA in patients with ventricular arrhythmia substrates and it is also questionable whether we can transfer the observations from atrial arrhythmias into patients with VAs.

In this context, Hoshiyama et al. demonstrated that slow CVV zones matched arrhythmia substrates in a patient with multiple VTs. The authors concluded that AMA might help to understand the relationship between electrograms and critical slow conduction areas, even in multiple and complex VTs [7].

Based on the reported findings mentioned above [1–3, 7], we hypothesized that decelerated CVV zones might indicate the anatomical location of the individual arrhythmia substrate inside the scar area or along the border zone. The correlation between CVV deceleration zones from AMA and areas of abnormal low-voltage from endocardial bipolar mapping was limited (37.5%). In contrast, we found a good correlation between scar from endocardial unipolar voltage mapping and CCV deceleration zones (94%).

A potential explanation for this finding can be the fact that unipolar voltage mapping is suggested to improve the visualization of scar tissue especially in the midmyocardial or epicardal layer. To create a global pattern of activation in the chamber of interest, coherent mapping uses anatomical data and LAT data using both unipolar and bipolar electrograms based on the Wavefront algorithm and thus can show areas of slow conduction in all layers of the myocardium.

Moreover, information from unipolar voltage mapping can visualize areas representing the extension and border of dense endocardial scar tissue resulting in an improved understanding of intramural or even epicardial exits of individual VT circuits [5, 6]. An advantage of the novel AMA is the integration of electrogram information from bi- and unipolar mapping at the same time, which might add valuable information in terms of intramural or epicardial VA substrates and mechanisms.

## Limitations

The present study is of observational design and therefore has typical limitations. The patient cohort is relatively small and not all patients received epicardial mapping. Nevertheless, this study is the first to provide information on CVV zones based on AMA in patients with VAs. This study was provided for assessment of the new AMA as an additional tool for better understanding of the VT substrate. Therefore, ablation strategy was not changed according to the AMA findings in this study cohort and will be investigated in further studies.

## Conclusion

The novel AMA improves the understanding of individual VA substrates due to the visualization of decelerated CVV zones and their correlation with abnormal low voltage predominantly from unipolar mapping. The utilization of AMA in conjunction with evidence for scar tissue might result in additional intramural or epicardial targets for VA ablation and improved freedom from arrhythmia recurrence.
